# Effects of valproate sodium on extracellular signal-regulated kinase 1/2 phosphorylation following hippocampal neuronal epileptiform discharge in rats

**DOI:** 10.3892/etm.2013.1343

**Published:** 2013-10-11

**Authors:** ZUCAI XU, JUN ZHANG, XIANZE LEI, ZHONGXIANG XU, YAN PENG, BENHAI YAO, PING XU

**Affiliations:** Department of Neurology, Affiliated Hospital of Zunyi Medical College, Zunyi, Guizhou 563003, P.R. China

**Keywords:** valproate sodium, extracellular signal-regulated kinase 1/2, neuron, epileptiform discharge

## Abstract

The aim of the present study was to investigate the effects of valproate sodium (VPAS) on the phosphorylation extracellular signal-regulated kinase 1/2 (ERK1/2) following hippocampal neuronal epileptiform discharge in rat neurons. The study used neurons from female and male neonate Sprague-Dawley (SD) rats (at least 24 h old), which were rapidly decapitated. Following the successful development of the epileptiform discharge cell model, the neurons were divided into two groups, the VPAS group and the control group. In the concentration-response experiment, the neurons were incubated with three different concentrations of VPAS (50, 75 and 100 mg/l) 30 min prior to the epileptiform discharge. The expression of phosphorylated ERK1/2 (p-ERK1/2) was examined using an immunofluorescence technique. In the time-response experiment, the neurons were incubated with VPAS (50 mg/l) and monitored at different time-points (30 min prior to the epileptiform discharge and 0 min, 30 min, 2 h and 6 h subsequent to epileptiform discharge), and western blotting was employed to measure the changes in p-ERK1/2 protein expression. No significant differences in the expression of p-ERK1/2 among the neurons treated with different concentrations of VPAS were identified in the concentration-response experiment. However, in the time-response experiment, the expression of p-ERK1/2 30 min prior to the epileptiform discharge was significantly lower compared with that at the other time-points. Furthermore, 50 mg/l VPAS was capable of decreasing the action potential frequency of the neuronal epileptiform discharge. ERK1/2 was excessively and persistently activated following the epileptiform discharge of the neurons. In addition, a low concentration of VPAS was effective at inhibiting the phosphorylation of ERK1/2 at an earlier period of neuronal epileptiform discharge.

## Introduction

It has previously been shown that extracellular signal-regulated kinase 1/2 (ERK1/2) is a member of the mitogen-activated protein kinase (MAPK) family, which is abundantly expressed in the neuron body and dendrites (1) Moreover, ERK1/2 acts as part of a signaling pathway activated by multiple upstream factors, such as growth factors, inflammatory factors, neurotransmitters and calcium influx, via the MAPK and ERK activator kinase (MEK). Furthermore, these two activator kinases are capable of phosphorylating ERK1/2 at threonine and tyrosine residues with the latter resulting in the dissociation of ERK1/2 from MEK1/2. Phosphorylated ERK1/2 (p-ERK1/2) enters the nucleus by passive diffusion of the monomer, active transport of the dimer or by a direct interaction of ERK1/2 with the nuclear pore complex, where it activates nuclear factors (2–5). Epilepsy is characterized by the excessively synchronized discharge of cerebral neurons and comprises a diverse group of syndromes with different etiologies. In epileptic seizures, normal brain tissue undergoes damage that produces permanent plasticity changes and results in epileptogenesis. Studies have revealed that in addition to ERK1/2 affecting neuronal excitability, mossy fiber sprouting and synaptic remodeling mechanisms are also involved in epileptogenesis (6–8).

Valproate sodium (VPAS) is a classical and broad-spectrum antiepileptic drug that is widely used in different types of epilepsy. VPAS predominantly exerts its antiepileptic effect through the γ-aminobutyric acid (GABA) system (9). In the present study, concentration- and time-response experiments were performed to measure the effect of VPAS on p-ERK1/2 expression, in order to provide an enhanced understanding of the cellular and molecular mechanisms underlying the antiepileptic effect of VPAS.

## Materials and methods

### Neuron culture and patch-clamp recording

Neonatal Sprague-Dawley (SD) rats, aged <24 h, were purchased from the Experimental Animal Center of Chongqing Medical University of China (Chongqing, China). Following anesthesia, the brain tissue was exposed and the bilateral hippocampi were excised using a dissecting microscope (Nikon Corp., Tokyo, Japan), prior to the meninges and superficial blood vessels being removed. The hippocampus was then minced in ice-cold D- Hank’s balanced salt solution (Sigma, St. Louis, MO, USA) and incubated in five-fold volumes of 0.125% parenzyme (Sigma) at 37ºC and in 5% CO_2_ for 20 min. The digestion was terminated by the addition of an equal volume of growth medium composed of Neurobasal^®^ Medium, 2% B-27, 0.5 mM L-glutamine and 0.5% fetal bovine serum (FBS; all from Gibco-BRL, Grand Island, NY, USA). Following centrifugation at 212 × g for 5 min, the supernatant was discarded and fresh growth medium was added. The tissue was then dissociated and the cell suspension was filtered through a 200-mesh cell sieve (Nanjing jing yu sheng instrument Co., Ltd., Nangjing, China). The cells were diluted to a concentration of 5×10^5^ cells/ml, plated on coverslips coated with polylysine (0.1%; Gibco-BRL) and maintained at 37ºC in 5% CO_2_. Twenty-four hours subsequent to plating, the medium was changed to a maintenance medium (Neurobasal Medium, 2% B-27, and 0.5 mM L-glutamine). Half the volume of the maintenance medium was changed every three days. Following nine days *in vitro*, cell excitability was measured using previously established procedures (6). Briefly, the cells were placed in magnesium-free artificial cerebrospinal fluid (ACSF; Chongqing chemical reagent factory, Chongqing, China) containing 124 mM NaCl, 3 mM KCl, 2 mM CaCl_2_, 2 mM MgCl_2_, 1.23 mM NaH_2_PO_4_, 26 mM NaHCO_3_, 10 mM glucose and 0.002 mM glycine (pH 7.3, 325 mOsm) for 3 h. To measure cell excitability, the whole-cell current-clamp technique was used to record the epileptiform activity. For whole-cell recording, the cultures were placed on the stage of an inverted microscope (Nikon Corp.) and continuously perfused with ACSF. Patch electrodes (2–4 MV resistance) were filled with the following internal solution: 60 mM K_2_SO_4_, 60 mM N-methyl-D-glucamine (NMG), 40 mM HEPES, 4 mM MgCl_2_, 0.5 mM 1,2-bis(2-aminophenoxy)ethane-N,N,N′,N′-tetraacetic acid (BAPTA), 12 mM phosphocreatine, 2 mM Na_2_ATP, 0.2 mM Na_3_GTP and 0.1 mM leupeptin (pH 7.2–7.3, 265–270 mOsm/l) (Sigma). At the zero holding current in the whole-cell model, the membrane potential was measured immediately. Neuronal recording was then performed using an Axon Axopatch 200B Capacitor Feedback Patch Clamp Amplifier (Axon Instruments, Inc., Molecular Devices Corp., Palo Alto, CA, USA) that was controlled and monitored using pCLAMP™ 9 Electrophysiology Data Acquisition and Analysis Software with an Axon DigiData 1320 Series Interface unit (Axon Instruments, Inc.).

### Immunofluorescence measurement of p-ERK1/2 in the concentration-response experiment

The wells of cultured neurons were randomly divided into control and VPAS groups. The control neurons displaying epileptiform activity were incubated in magnesium-free medium for 3 h and were examined at 0 min. The VPAS-treated neurons were incubated in magnesium-free ACSF to which VPAS (50, 75 and 100 mg/l, respectively) was added 30 min prior to the epileptiform discharge, and then were examined at time-points corresponding with those of the control group. p-ERK1/2 levels were detected absolutely using immunofluorescence. Briefly, the cultured neurons were washed with phosphate-buffered saline (PBS) for 3–5 min, fixed in 4% paraformaldehyde for 30 min and washed with PBS for 3–5 min. The neurons were then treated with 0.5% Triton X-100 (Gibco-BRL) for 20 min at room temperature, washed with PBS for a further 3–5 min and blocked with 10% goat serum for 20 min at room temperature. The neurons were subsequently incubated with mouse anti-p-ERK1/2 (1:100; Santa Cruz Biotechnology, Inc., Santa Cruz, CA, USA) overnight at 4ºC. Secondary antibodies [goat anti-mouse-fluorescein isothiocyanate (FITC), 1:50; Santa Cruz Biotechnology, Inc.] were applied for 2 h at room temperature, prior to the neurons being washed with PBS for 3–5 min. Images of each sample were captured using a laser scanning confocal microscope (Leica Microsystems, Wetzlar, Germany).

### Western blot analysis of p-ERK1/2 in the time-response experiment

The neurons were divided into two groups, identical to those in the concentration-response experiment. The levels of p-ERK1/2 in the control and VPAS (50 mg/l) groups were measured using western blotting at different time-points (30 min prior to epileptiform discharge and 0 min, 30 min, 2 h and 6 h subsequent to epileptiform discharge). The cultured neurons were washed with cold PBS, collected using centrifugation (11,190 × g for 5 min) and lysed in cell lysis buffer (100 ml) containing Tris-HCl (50 mM; pH 8.0), NaCl (150 mM), EDTA (1 mM), ethylene glycol-O,O′-bis(2-aminoethyl)-N,N,N′,N′-tetraacetic acid (EGTA; 1 mM), Triton X-100 (1%), phenylmethylsulfonyl fluoride (1 mM) and a freshly added protease inhibitor cocktail (Calbiochem, La Jolla, CA, USA) on ice for 30 min. The protein (50 mg) was then resolved on a 10% polyacrylamide gel, transferred to a polyvinylidene difluoride (PVDF) membrane and blocked for 1 h at room temperature with 5% nonfat dried milk in PBS. The membranes were subsequently incubated with primary antibodies (mouse anti-p-ERK1/2 at 1:1,000 or rabbit anti-β-actin at 1:2,000; Santa Cruz Biotechnology, Inc.) in blocking buffer. The blots were washed for 3–10 min each with PBS plus Tween-20 (0.1%) and then incubated with the appropriate diluted horseradish peroxidase (HRP)-tagged secondary antibody (1:1,000; Santa Cruz Biotechnology, Inc.) for 1 h at room temperature. The blots were developed in accordance with the manufacturer’s instructions using SuperSignal™ West Pico Chemiluminescent HRP substrate (Pierce Protein Biology Products, Thermo Fisher Scientific, Inc., Rockford, IL, USA), and visualized following exposure to X-ray film. The band intensities were calculated using the GelWorks 4.1 image analysis system (UVP products, Upland, CA, USA).

### Statistical analysis

Data are presented as the mean ± standard deviation (SD), and SPSS version 18.0 statistical software (SPSS, Inc., Chicago, IL, USA) was used for the statistical analysis. Significant differences between the groups were assessed using one-way analysis of variance (ANOVA). P<0.05 was considered to indicate a statistically significant difference.

## Results

### Effects of different concentrations of VPAS on the phosphorylation of ERK1/2 following hippocampal neuronal epileptiform discharge

**Figure 1:**
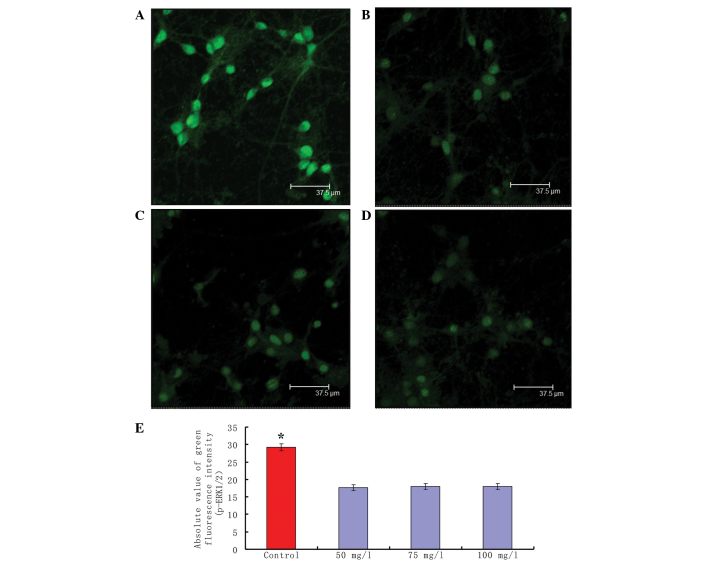
Phosphorylated extracellular signal-regulated protein kinase 1/2 (p-ERK1/2) labeled by immunofluorescence. (A) Control group; (B) 50 mg/l valproate sodium (VPAS); (C) 75 mg/l VPAS; (D) 100 mg/l VPAS. (E) The absolute value of the green fluorescence intensity in the control and VPAS groups. ^*^P<0.01 compared with the VPAS groups (n=20).

Significant differences were observed in the expression of p-ERK1/2 between untreated and VPAS-treated hippocampal neurons displaying epileptiform discharge, as assessed using immunofluorescence labeling. In the control neurons, a high intensity of green fluorescence, reflecting a high level of p-ERK1/2, was observed in the nucleus and cytoplasm (Fig. 1A). However, decreased p-ERK1/2 green fluorescence intensity was apparent in the VPAS-treated neurons. VPAS treatment 30 min prior to discharge at different concentrations (50, 75 and 100 mg/l) significantly inhibited the green fluorescence intensity of p-ERK1/2 in the hippocampal neurons compared with that in the control group, (Fig. 1B–D). Moreover, the phosphorylation level of ERK1/2 did not vary with the change in VPAS concentration. A total of 20 neurons were randomly selected from each group and a comparison of the absolute fluorescence intensity of p-ERK1/2 staining between the control and VPAS groups was performed. Statistical analysis using the Student’s t-test revealed significant differences in the fluorescence intensity of p-ERK1/2 staining between the untreated and VPAS-treated hippocampal neurons (P<0.01; Fig. 1E).

### Effects of VPAS on the phosphorylation of ERK1/2 at different time-points following hippocampal neuronal epileptiform discharge

**Figure 2:**
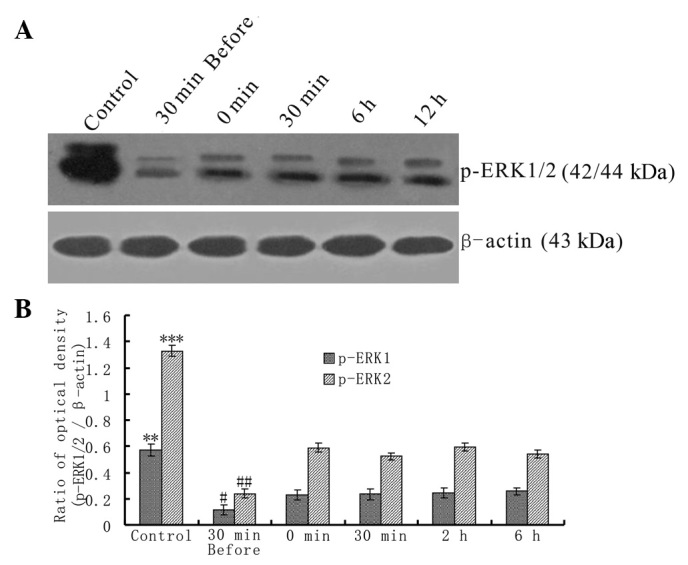
Phosphorylated extracellular signal-regulated protein kinase 1/2 (p-ERK1/2), examined using western blotting. (A) The lanes labeled 30 min before, 0 min, 30 min, 6 h and 12 h represent time-points in the valproate sodium (VPAS) group (50 mg/l), while Control represents the control group. (B) The optical density ratio of p-ERK1/2 to β-actin: ^**^P<0.01 (p-ERK1), ^***^P<0.01 (p-ERK2) compared with the VPAS group (n=5); ^#^P<0.01 (p-ERK1), ^##^P<0.01 (p-ERK2) compared with the other four time-points (n=5).

The protein levels of p-ERK1/2 in the untreated and VPAS-treated neurons were examined using western blotting. Measurements of p-ERK1/2 levels in untreated neurons were made following the culture of the neurons with magnesium-free ACSF for 3 h (Fig. 2A, Control). The expression level of p-ERK1/2 was significantly reduced at all time-points in the VPAS-treated neurons (Fig. 2A, 30 min before, 0 min, 30 min, 6 h and 12 h) compared with the expression level in the control group. The lowest expression of p-ERK1/2 was observed at the ‘30 min before’ time-point. The relative expression levels of p-ERK1/2 in the two groups were divergent at all the examined time-points (P<0.01).

### Effects of VPAS on action potential frequency following hippocampal neuronal epileptiform discharge

**Figure 3:**
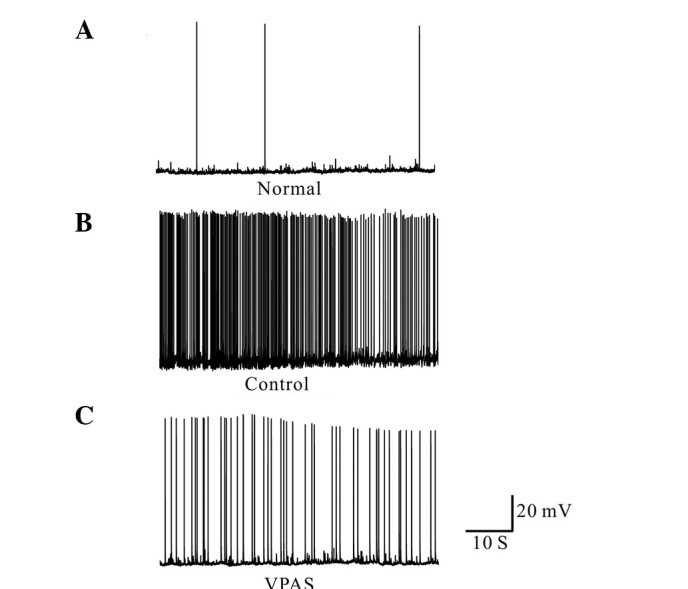
Valproate sodium (VPAS) attenuates magnesium-free induced epileptiform activity in cultured hippocampal neurons. (A) Recording from a normal neuron displaying baseline activity, consisting of intermittent action potentials. (B) Induction of continuous epileptiform activity in a neuron upon magnesium-free treatment. (C) VPAS (50 mg/l) attenuates magnesium-free induced epileptiform activity.

Current recordings from the normal neurons displayed occasional action potentials, as shown in Fig. 3A (Normal). By contrast, culturing the neurons in magnesium-free medium for 3 h resulted in the development of continuous, high-frequency burst discharges that evolved into recurrent epileptiform discharges. The spike frequency of these neurons was >3 Hz. This epileptiform discharge was continuous throughout the 60 min of recording (Fig. 3B). At 15 min following 50 mg/l VPAS perfusion, the spike frequency was significantly decreased (Fig. 3C).

## Discussion

Treatment of rat neurons with three different concentrations of VPAS 30 min prior to epileptiform discharge in magnesium-free ACSF blocked the expression of p-ERK1/2. No significant differences were identified among the phosphorylation levels of the neurons treated with 50, 75 and 100 mg/l VPAS. This result suggested that the effect of VPAS was not concentration-dependent. Furthermore, following the incubation of the neurons with 50 mg/l VPAS and the analysis of p-ERK1/2 levels at five different time-points, it was observed that the lowest expression of p-ERK1/2 was at ‘30 min before’ and the downward trend was continued at 0 min, 30 min, 2 h and 6 h subsequent to epileptiform discharge. It was observed that 50 mg/l VPAS was capable of significantly decreasing the epileptiform activity. The study therefore demonstrated that ERK1/2 signaling acted as a downstream target of VPAS in the regulation of epilepsy.

MAPKs belong to a large family of proline-directed serine-threonine protein kinases that are fundamental in cellular functions. MAPKs include ERK1/2, ERK3/4, ERK5, ERK7/8, c-Jun N-terminal kinase (JNK)-1/2/3 and p38 (α/β/γ/δ) MAPK (10–12). The activation of MAPK proceeds through a cascade of upstream molecules in an orderly fashion, with activation occurring at a low level in certain MAPKs and particularly in ERK1/2, which is most likely due to basal signaling and metabolic needs (13). The nature of the upstream molecules depends on the stimulatory trigger, cell type and the subcellular location of activation. The activation of MEK1/2 leads to the phosphorylation of threonine and tyrosine residues of ERK1/2, with recognition sites of Thr-Glu-Tyr (TEY). ERK1 and ERK2 are homologous isoforms that share the same substrate specificities *in vitro*. These two proteins, which phosphorylate a multitude of protein substrates, have ~85% amino acid identity and are expressed in almost all tissues with much greater identity in the core regions (14). Studies focusing on the extracellular signal transduction pathway are likely to enhance the understanding of epilepsy with new theories and technologies. Using *in vitro* and *in vivo* epilepsy models, it has been demonstrated that ERK1/2 is involved in the occurrence and development of epilepsy. The phosphorylation of ERK1/2 was shown to be significantly enhanced in a kainic acid mouse model (15). Furthermore, in a pilocarpine-evoked model, ERK1/2 was rapidly activated, particularly in the hippocampal dentate gyrus granule cells, and the activation was completed prior to the seizure (8). In our previous study, it was observed that there was marked expression of p-ERK1/2 in epileptic neurons, and the level of expression was demonstrated to be greater than that in normal neurons. Moreover, the phosphorylation level of ERK1/2 peaked at 30 min following epileptiform discharge (6). The study showed that the expression of p-ERK1/2 was marked in the neurons at the end of the 3-h treatment with magnesium-free ACSF. In an epileptic model evoked by the potassium channel inhibitor 4-aminopyridine, the peak phosphorylation level of ERK1/2 was observed at 20 min. Pretreatment with an inhibitor of ERK1/2 inhibited ERK1/2 phosphorylation and also blocked the epileptiform discharge completely during the ictal period (16). In the fragile X syndrome mouse model simulated using gene knockout technology, ERK1/2 was observed to participate in group I metabotropic glutamate receptor (I mGluR)-mediated epileptiform discharge (17). In addition, the expression of p-ERK1/2 was shown to be notable in the temporal lobe and hippocampus of patients with drug-resistant epilepsy (18). It has been shown that the phosphorylation of EKR1/2 is one of the early cell responses in seizures, and may therefore be regarded as a potential therapeutic target to prevent chronic epilepsy (19).

VPAS, a broad-spectrum and first-line antiepileptic drug, is capable of controlling most types of seizures, including absence, myoclonic and generalized tonic-clonic seizures, as well as status epilepticus. The primary antiepileptic pharmacological effect of VPAS is to inhibit the GABA enzyme and succinic semialdehyde dehydrogenase. In order to increase the concentration of GABA in the brain, VPAS is also able to inhibit N-methyl-D-aspartate (N-methyl-D-aspartic acid, NMDA) receptor-mediated neuron depolarization, and to inhibit the Ca^2+^ influx leading to K^+^ conduction (20). The results of the present study suggest that VPAS decreases the action potential frequency induced by magnesium-free ACSF. However, its potential and multiple mechanisms have not yet been fully elucidated. An investigation into VPAS and the signal transduction pathway reported that VPAS was capable of significantly reducing protein kinase C (PKC) and G protein activity, with specificity for α and ɛ moieties (9). Tang *et al*(21) demonstrated that PKC small interfering RNA (siRNA) completely inhibited acetylcholine-induced mesenchymal stem cell migration by blocking ERK1/2 phosphorylation (21). Furthermore, the PKC pathway has been shown to protect LNCaP prostate cancer cells from phorbol ester-induced apoptosis by promoting ERK1/2 (22). The results of the present study indicate that the enhancement of ERK1/2 phosphorylation following epileptiform discharge is significantly decreased by VPAS in primary cultured hippocampal neurons. The results showed notable timeliness, with a low-dose effective concentration of VPAS inhibiting the phosphorylation of ERK1/2 at an earlier period of neuronal epileptiform discharge. The negative regulatory mechanism of VPAS on the signal transduction pathway has yet to be elucidated. Studies have shown that brain-derived neurotrophic factor (BDNF) is an activator of ERK1/2, and that its upregulation may evoke excessive neuronal excitability and trigger mossy fiber sprouting (23,24). Future studies of the specific mechanisms are required.

In conclusion, the association between VPAS and the cell signal transduction pathways is complex and diverse, and the effect of VPAS on the level of ERK1/2 phosphorylation is only one of numerous factors. The results of the present study have provided a new experimental basis for further investigation into the mechanism underlying the VPAS-induced suppression of seizure-onset. This may facilitate the identification of a novel target for the development of future anticonvulsant therapies.
